# Novel mitochondrial targeting charge-reversal polysaccharide hybrid shell/core nanoparticles for prolonged systemic circulation and antitumor drug delivery

**DOI:** 10.1080/10717544.2019.1687614

**Published:** 2019-11-18

**Authors:** Lei Fang, Wei Zhang, Zhen Wang, Xinxin Fan, Ziting Cheng, Xiaoya Hou, Daquan Chen

**Affiliations:** a Collaborative Innovation Center of Advanced Drug Delivery System and Biotech Drugs, Universities of Shandong, Yantai University, Yantai, PR China;; b Department of Radiotherapy, Affiliated Yantai Yuhuangding Hospital of Qingdao University, Yantai, PR China

**Keywords:** Tumor microenvironment-responsive, long-circulating, charge-reversal, polysaccharide-based nanoparticles, mitochondrial targeting shell/core structure

## Abstract

Stability in systemic circulation, effective tumor accumulation, and the subsequent crucial subcellular targeting are significant elements that maximize the therapeutic efficacy of a drug. Accordingly, novel nanoparticles based on polysaccharides that simultaneously presented prolonged systemic circulation and mitochondrial-targeted drug release were synthesized. First, the mitochondrial-targeted polymer, 3,4-dihydroxyphenyl propionic acid-chitosan oligosaccharide-dithiodipropionic acid-berberine (DHPA-CDB), was synthesized, which was used to form self-assembled curcumin (Cur)-encapsulated cationic micelles (DHPA-CDB/Cur). Negatively charged oligomeric hyaluronic acid-3-carboxyphenylboronic acid (oHA-PBA), a ligand to sialic acid and CD44, was further added to the surface of the preformed DHPA-CDB/Cur core to shield the positive charges and to prolong blood persistence. oHA-PBA@DHPA-CDB/Cur formed a covalent polyplex of oHA-PBA and DHPA-CDB/Cur via the pH-responsive borate ester bond between PBA and DHPA. The mildly acidic tumor environment led to the degradation of borate ester bonds, thereby realizing the exposure of the cationic micelles and causing a charge reversal from −19.47 to +12.01 mV, to promote cell internalization and mitochondrial localization. Compared with micelles without the oHA-PBA modification, the prepared oHA-PBA@DHPA-CDB/Cur showed enhanced cytotoxicity to PANC-1 cells and greater cellular uptake via receptor-mediated endocytosis. oHA-PBA@DHPA-CDB/Cur was effectively targeted to the mitochondria, which triggered mitochondrial membrane depolarization. In mice xenografted with PANC-1 cells, compared with control mice, oHA-PBA@DHPA-CDB/Cur resulted in more effective tumor suppression and greater biosafety with preferential accumulation in the tumor tissue. Thus, the long-circulating oHA-PBA@DHPA-CDB/Cur, with mitochondrial targeting and tumor environment charge-reversal capabilities, was shown to be an excellent candidate for subcellular-specific drug delivery.

## 1. Introduction

The tumor microenvironment (TME), which contains complex stroma cells and matrix components, is believed to be a major cause of the difficulties facing the delivery of nanomedicines (Roma-Rodrigues et al., 2019). Many features of TME discriminate it from normal tissues (Gulzar et al., 2019), such as particular enzymes (e.g. matrix metalloproteinases [MMPs]) (Ma et al., 2019; Zhang et al., 2019) higher glutathione (GSH) levels (Xue et al., 2019), lower pH values(Hu et al., 2017; Jiang et al., 2018), hypoxic conditions (Wang et al., 2017). Given the extreme difficulty of delivering an ample payload that will reach and enter the tumor tissue, several multifunctional polymer nanoparticles with antitumor effects, which are sensitive to the TME, have attracted attention in the field of antitumor drug delivery (Lang et al., 2019; Taleb et al., 2019).

The major challenges that present tremendous barriers to the delivery of polymeric nanoparticles from the initial injection site to the final tumor site, such as the reticuloendothelial system (RES) barriers and nonspecific cellular internalization, have ultimately reduced the therapeutic efficacy of polymeric nanoparticles (Ling et al., 2018; Seidi et al., 2018; Zhang et al., 2018). In particular, cationic polymer nanoparticles, which are able to promote cellular internalization and mitochondrial location, are usually strongly cytotoxic, have poor serum stability, and are easily removed by the RES (Borri et al., 2018; Zhou et al., 2017). To prevent these problems and prolong the blood persistence *in vivo*, a negatively charged shell of polyethylene glycol (PEG) (Ranalli et al., 2017; Chuang et al., 2017) and polysaccharides (e.g. hyaluronic acid) (Wang et al., 2016, 2019a) is usually applied. Subsequently, TME-responsive charge reversal can be triggered based on the differences in the physiological environment (e.g. pH or enzyme content) (Chen et al., 2017; Jing et al., 2018; Xu et al., 2017) between normal tissue and tumor tissue. Further, the ligand-targeting capability can be modified to enhance the ability of the polymer nanoparticles to specifically identify tumor cells (Dai et al., 2018).

Mitochondrially targeted polymeric nanoparticles have recently attracted much attention as an ideal subcellular target in tumor-targeted therapy (Lv et al., 2018; Pan et al., 2018). Compared with the mitochondria in normal cells, the mitochondria in tumor cells exhibit many differences (Babu et al., 2017), such as high levels of reactive oxygen species (ROS) (Zheng et al., 2017), a reducing environment (e.g. due to GSH) (Besson et al., 2019), and higher membrane potential (Δψm) (Wang et al., 2019b). In addition to conventional mitochondrial-targeted groups (e.g. triphenylphosphonium, TPP) (Sun et al., 2017; Yang et al., 2018), berberine (Ber) derivatives have been reported as an effective mitochondrial-targeting substance owing to their positively charged center and strong lipophilicity(Song et al., 2019; Tuo et al., 2016). Furthermore, mitochondria have been demonstrated as a vital organelle used for the regulation of the cellular apoptosis pathway and to trigger cell death. The pathways of apoptosis include the depolarization of the mitochondrial membrane, which leads to the release of cytochrome C and other apoptotic proteins (e.g. caspase 3) (Tan et al., 2018; Zhou et al., 2018).

Phenylboronic acid (PBA), a tumor-targeting ligand, can be selectively bound to the sialic acid epitopes that are overexpressed on the surface of various tumor cells (Kundu et al., 2019). Therefore, the tumor-targeting ability of a nanocarrier can be increased when the carrier is combined with PBA (Jeong et al., 2017; Kim et al., 2016). In addition, it is notable that PBA can selectively and reversibly combine with chemical compounds (e.g. glucose, fructose, adenosine triphosphate [ATP], and DHPA) containing cis-o-diol or meta-diol structure to form reversible borate esters (Song et al., 2019; Zhou et al., 2018). The borate ester bond is highly susceptible to certain conditions including ATP, pH, and ROS; this rare property has ensured that thr PBA structural unit is used widely in TME-responsive nanosystems (Fan et al., 2017; Huang et al., 2019).

**Figure 1.**
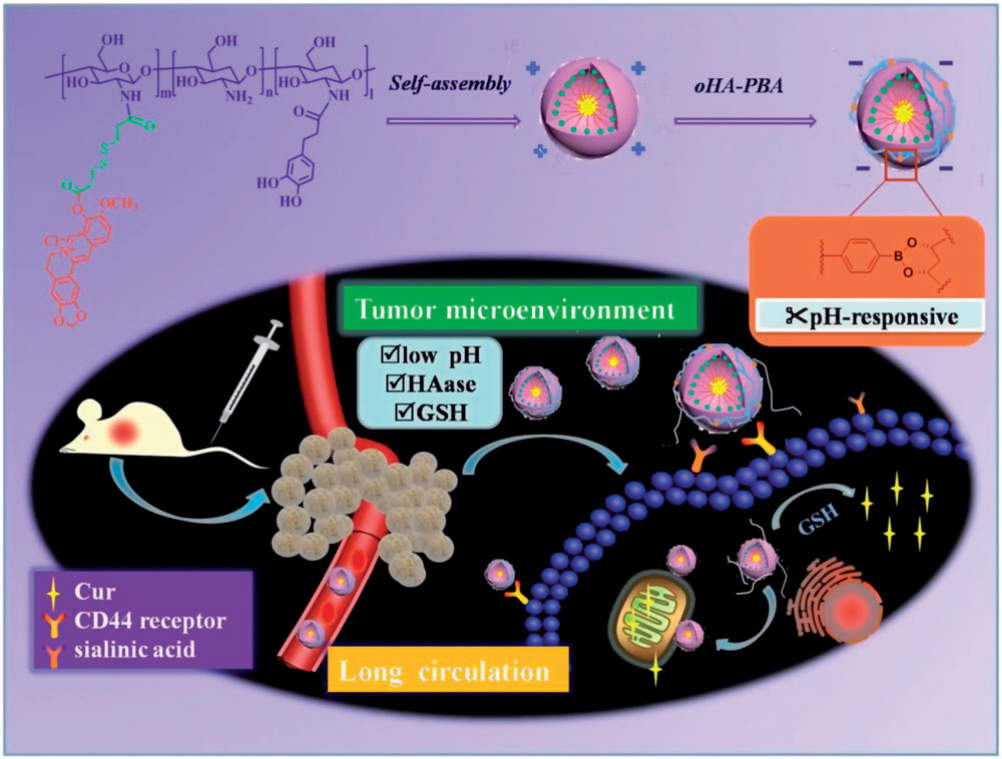
Schematic illustration of oHA-PBA-coated DHPA-CDB/Cur (oHA-PBA@DHPA-CDB/Cur) used to TME-rsponsive and mitochondrially targeted antitumor drug delivery.

Based on the above review, in our study, we prepared nanoparticles based on polysaccharides that simultaneously displayed prolonged systemic circulation, mitochondrially targeted drug release, and TME-responsive charge reversal ability. The system was constructed from the mitochondrial-targeted core of DHPA-CDB/Cur with a negatively charged oHA-PBA surface modification. oHA-PBA@DHPA-CDB/Cur formed a covalent polyplex from oHA-PBA and DHPA-CDB/Cur via the pH-responsive borate esters bond between PBA and DHPA. Compared with electrostatic bonding, covalent polyplex bonding can overcome the disadvantage whereby physical binding is easily replaced by physiological anions (e.g. phosphate), and have excellent stability. Simultaneously, oHA-PBA@DHPA-CDB/Cur was designed to achieve the specific targeting and be endocytosed by the tumor cells through the specific binding of the sialic acid epitope (Huo et al., 2018) and the CD44 receptor, which are overexpressed on the tumor cells. When entering into the TME by receptor-mediated targeting, this nanosystem would turn the positive charge into the negative charge, promoting cellular internalization and mitochondrial location, ultimately achieving GSH-triggered Cur release. In this work, we have examined the elementary physicochemical properties of DHPA-CDB/Cur and oHA-PBA@DHPA-CDB/Cur, including correlative characterization and TME-responsive drug release. The *in vitro* cytotoxicity, cellular uptake, mitochondrial location, and depolarizing effect on the mitochondrial membrane of DHPA-CDB/Cur and oHA-PBA@DHPA-CDB/Cur on PANC-1 cells were investigated by using the MTT assay and an inverted fluorescence microscope. Model mice xenografted with PANC-1 cells were established, and the *in vivo* pharmacodynamics and imaging were evaluated in this model (Figure 1).

## 2. Experimental Section

### 2.1. Materials

COS (MW 322-1610) was obtained from Weikang Biopharmaceutical Co., Ltd (Shandong, China). oHA (MW <10 KDa) was selected from Freda Co. Ltd (Shandong). Berberine chloride and Cur were bought from Tipao Biotechnology Co., Ltd (Shanghai). EDC, DMAP, NHS, oxalyl chloride DHPA, PBA, HAase, anhydrous tetrahydrofuran, and oxalyl chloride were all purchased from Aladdin Reagent Net. 3,3-dithiodipropionic acid was obtained from Macklin Reagent Co, Ltd. Triethylamine (TEA) was prepared by Tianjin Fuyu Industrial Corporation.

MTT, DMEM, and PBS powder packages were obtained from Saiersi Biotechnology Co. Ltd (Shangdong); FBS, Hoechst 33342, Lyso-Tracker red, and JC-1 were obtained from Beyotime Net; Mitotracker Red CMXRos was purchased from Meilun Biotechnology Co., Ltd (Dalian, China); and DiR iodide was obtained from RuiTaibio (Beijing, China). Female BALB/c nude mice were obtained from Vital River Laboratory Animals Technology Co., Ltd (Beijing, China).

### 2.2. Positively charged carrier material synthesis

#### 2.2.1. Synthesis of DHPA-COS

3,4-Dihydroxyphenyl propionic acid-grafted chitosan oligosaccharide (DHPA-COS) was prepared by an amidation reaction. Briefly, 90 mg COS and 50 mg DHPA were mixed in deionized water containing EDC and NHS, and stirred at RT for 6 h. To remove the unreacted reactants and catalysts by dialysis (MWCO 300 Da), the mixture was dialyzed for 24 h in deionized water and the sink solution was regularly replaced. The final product was obtained by lyophilization.

#### 2.2.2. Synthesis of DHPA-CDB

**Figure 2.**
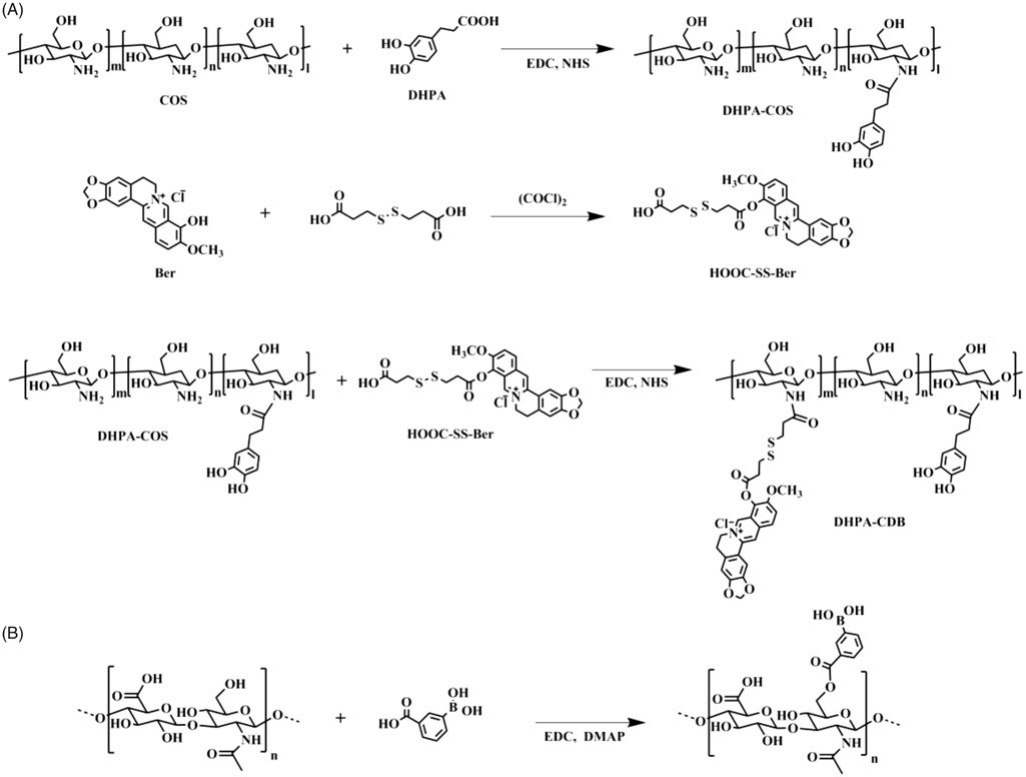
Synthetic scheme of polymers: (A) DHPA-CDB. (B) oHA-PBA.

The synthesis steps of 3,4-dihydroxyphenyl propionic acid-Chitosan oligosaccharide-Dithiodipropionic acid-Berberine (DHPA-CDB) are shown in Figure 2.

First, to produce the active berberine (Ber) groups, berberine chloride was placed in a vacuum container at 190 °C–210 °C for 15 min; the product was purified by column chromatography to obtain dark red berberrubine.

Second, to synthesize dithiodipropionic acid-Berberine (HOOC-SS-Ber): in anhydrous tetrahydrofuran, the ratio of the input of oxalyl chloride was controlled, and one end of the dithiodipropionic acid was activated to form an acyl chloride. The detailed procedure was as described below: in an ice bath, 60 μL oxalyl chloride was added to 1 mL anhydrous tetrahydrofuran solution containing 50 mg of dithiodipropionic acid while stirring. After a while, the mixture was warmed to 35 °C and held at a constant temperature for 2 h to continue the reaction. Finally, the product was achieved by rotary evaporation. This product and Ber were completely dissolved in anhydrous tetrahydrofuran; subsequently, a catalytic amount of triethylamine was added, and the reaction was continued at 45 °C for 4 h. The homogenous product HOOC-SS-Ber was obtained by using column chromatography.

Finally, for the synthesis of DHPA-CDB: HOOC-SS-Ber was completely dissolved in DMSO and placed in a reaction vessel; EDC and NHS were added dropwise to activate HOOC-SS-Ber for 2 h. Then, the activated HOOC-SS-Ber was added to the freeze-dried product, DHPA-COS, and heated to 45 °C. After 48 h, the reaction mixture was poured into a dialysis bag (MWCO 300 Da) and purified in deionized water for 48 h in the dark. After dialysis, the solution in the dialysis bag was centrifuged, and the supernatant was lyophilized to obtain the end-product, DHPA-CDB.

### 2.3. Synthesis of oHA-PBA

The synthesis steps of 3-carboxyphenylboronic acid-grafted oligomeric hyaluronic acid (oHA-PBA) are shown in Figure 2. PBA was conjugated with oHA using EDC and DMAP as catalytic agents. Briefly, PBA (21 mg) was dissolved in formamide, and EDC and DMAP were added dropwise to activate PBA for 2 h. Then, oHA (50 mg) in formamide was added dropwise to the above solution, and stirred for 24 h at 55 °C. To remove the unreacted reactants and catalysts by dialysis, the solution was dialyzed (MWCO 2000 Da) for 24 h in deionized water, which was regularly replaced. The final product was obtained by lyophilization.

### 2.4. Preparation of DHPA-CDB/cur

Cur-encapsulated DHPA-CDB micelles (DHPA-CDB/Cur) were prepared by a thin-film hydration method. Cur (1 mg) and DHPA-CDB (10 mg) were co-dissolved in methanol, and the organic solvent was removed by rotary evaporation. After the film was formed, 3 mL deionized water was added; the mixture was hydrated for 1 h and then incubated at 60 °C for 2 h. The ultrasound was continued until the film membrane on the wall was completely dispersed in the water. The mixture was centrifuged and filtered through a microporous filter to obtain DHPA-CDB/Cur.

### 2.5. Preparation of oHA-PBA@DHPA-CDB/cur

The method of simple mixing was used to prepare the oHA-PBA-coated DHPA-CDB/Cur (oHA-PBA@DHPA-CDB/Cur), as described below. In the presence of vigorous stirring, freeze-dried HA-PBA (0.75 mg) powder was added to DHPA-CDB/Cur (3 mL), and reacted for 8 h at RT.

### 2.6. Characterization of DHPA-CDB/cur and oHA-PBA@DHPA-CDB/cur

The size, size distribution, polydispersity index (PDI), and zeta potential of the Cur preparations were determined by using a Beckman Coulter Particle Analyzer. The morphology of DHPA-CDB/Cur and oHA-PBA@DHPA-CDB/Cur was examined by using transmission electron microscopy (TEM). The Cur standard solution was prepared in acetonitrile and a standard curve was constructed to calculate the amount of Cur in each Cur preparation. The drug loading (DL, %) and encapsulation efficiency (EE, %) of the Cur preparations were calculated. DL is the percentage of Cur content and represents the total quality of the preparation. EE is the percentage of the measured Cur loading relative to the theoretical Cur loading.

To demonstrate the existence of chemical cross-links between DHPA and PBA, the stability of oHA-PBA@DHPA-CDB/Cur in physiological ion conditions was evaluated from the measurement of the changes in particle size over a specified time interval. We used PBS to simulate physiological ion conditions. oHA-PBA@DHPA-CDB/Cur was diluted with pH 7.4 PBS buffer and placed at 4 °C. The particle sizes were measured at 0, 4, 8, 12, 24, 48, and 72 h after the solution was prepared.

### 2.7. Abscission of oHA-PBA from oHA-PBA@DHPA-CDB/cur at different pH values

The pH-mediated abscission of oHA was determined from the changes in surface charge of oHA-PBA@DHPA-CDB/Cur at different pH values (7.4, 6.5, and 5.0) in the presence or absence of hyaluronidase (HAase). After exposure for 2 h, the samples were extracted and their zeta potential was measured.

### 2.8. TME-responsive drug release

To examine the TME-responsive release characteristic of Cur from DHPA-CDB/Cur and oHA-PBA@DHPA-CDB/Cur, the release profiles were examined by using dialysis. First, we examined the GSH-responsive Cur release capacity of the DHPA-CDB/Cur core. Briefly, 1 mL DHPA-CDB/Cur was placed in a dialysis bag (MWCO 2000 Da) and immersed in 45 mL PBS buffer containing 0.5% (v:v) Tween 80 with different GSH concentrations (0.1, 1, and 10 mM). The whole system was agitated at 37 °C and sampled at pre-defined time points. The samples were were filtered through a 0.22 μm microporous filter and the concentration of Cur in the samples was determined by HPLC in triplicate. Subsequently, the *in vitro* Cur release kinetics from oHA-PBA@DHPA-CDB/Cur at various pH values (7.4, 6.5, and 5.0) and GSH (10 mM) were examined by using the same experimental method as above.

### 2.9. Cell culture

The complete growth medium, which was used to culture and expand pancreatic epithelioid carcinoma cells (PANC-1), was Dulbecco’s Modified Eagle Medium (DMEM) cell culture medium supplemented with 10% (v/v) fetal bovine serum (FBS). The PANC-1 cells were cultured in an incubator with an atmosphere of 5% CO_2_.

### 2.10. Cytotoxicity assay

The effects of Cur preparations on PANC-1 cells *in vitro* was examined by using an MTT assay. To compare the viability effects, free Cur, DHPA-CDB/Cur, and oHA-PBA@DHPA-CDB/Cur were fully dissolved in DMEM for final Cur concentrations from 2.5 μg/mL to 40 μg/mL. Typically, after counting, PANC-1 cells were uniformly dispersed in cell culture medium at a density of 50,000 cells/mL, and 200 μL of the cell suspension was placed in each well of a 96-well plate, and cultured overnight until the cells were fully adherence. Subsequently, fresh DMEM-containing Cur preparations were replaced and incubated for an additional 24 or 48 h. The microplate reader was conducted to gauge the relative cell viability.

To verify the targeting effect of the oHA-PBA, PANC-1 cells were first cultured in the presence or absence oHA-PBA (5 mg/mL) for 4 h. Subsequently, to negate the effect of oHA on the growth of PANC-1 cells, the experiment was conducted at high Cur concentrations. Cells were co-incubated with oHA-PBA@DHPA-CDB/Cur (10–40 μg/mL) for another 24 h and the *in vitro* cell viability of PANC-1 cells was examined by using the MTT assay.

### 2.11. Subcellular drug distribution

To reveal the subcellular drug distribution of micelles in PANC-1 cells, an inverted fluorescence microscope was used to obverse the intracellular distribution of Cur when supplied as free Cur, DHPA-CDB/Cur, and oHA-PBA@DHPA-CDB/Cur. PANC-1 cells, at a density of 3.8×10^5^ cells per well, were placed in 6-well plates and allowed to adhere for 48 h. Subsequently, the cell medium was then replaced with fresh medium containing Cur or Cur preparations (20 μg/mL) for 4 h. After removal of the Cur-containing medium, the cells were washed three times with PBS and incubated with MitoTracker Red CMXRos (25 nM) for 30 min. Finally, the cells were stained with Hoechst 33342 (10 μg/mL) for 15 min. After washing with PBS, the cells were fixed and observed under a microsccope.

### 2.12. *In vitro* cellular uptake and mitochondrial co-localization

To examine the cellular uptake and mitochondrial-targeting capacities of free Cur, DHPA-CDB/Cur, and oHA-PBA@DHPA-CDB/Cur at 20 μg/mL Cur, PANC-1 cells were cultured in 6-well plates and were subjected to various treatments. After incubation with the formulations for different times (1 h, 2 h, and 4 h), PBS was used to wash off residual Cur on the cell surface and the mitochondria were stained with MitoTracker Red CMXRos (25 nM) for 30 min. After washing, the cells were observed by using an inverted fluorescence microscope.

We also examined the concentration dependence of cellular uptake. Free Cur, DHPA-CDB/Cur, and oHA-PBA@DHPA-CDB/Cur were completely dissolved in DMEM to final Cur concentrations of 5 μg/mL, 15 μg/mL, and 20 μg/mL, respectively. The cells were washed, fixed, and observed by using an inverted fluorescence microscope.

### 2.13. Cellular mitochondrial membrane depolarization assay

The cationic fluorochrome JC-1 was selected as the specific fluorescent dye to evaluate the changes in Δψm; the conversion from red fluorescence to green fluorescence was observed by using an inverted fluorescence microscope. PANC-1 cells, at a density of 1.6×10^5^ cells/well, were seeded in 12-well plates, allowed to adhere to the plate, and grown for 48 h. Then, after incubation with free Cur, blank DHPA-CDB micelles, DHPA-CDB/Cur, and oHA-PBA@DHPA-CDB/Cur at 20 μg/mL Cur for 4 h, the cells were imaged. Untreated cells were used as the control group and treatment with CCCP was used as a positive control to induce the complete loss of Δψm.

### 2.14. Establishment of animal model

All animal studies were conducted in accordance with nationally established standards. PANC-1 cells were hypodermically injected in the right anterior position of nude mice (6–8 weeks old). When the tumor volume reached 70 mm^3^, the experiments were performed.

### 2.15. *In vivo* distribution of mice xenografted with pancreatic cancer cells

An *in vivo* small animal imaging system was used to investigate the bio-distribution of DiR-loaded DHPA-CDB micelles (DHPA-CDB/DiR) and oHA-PBA-coated DHPA-CDB/DiR micelles (oHA-PBA@DHPA-CDB/DiR) in nude mice bearing PANC-1 cells. Free DiR (as a control) and the DiR preparation were injected intravenously into the mice. The mice were anesthetized and photographed at a predetermined time points (2 h, 4 h, 8 h, 12 h, and 24 h). To investigate the distribution of DiR in each organ, the mice were sacrificed at 4 h after administration and the major visceral organs, including the heart, liver, spleen, lung, and kidney, and the tumor were captured by using the *in vivo* small animal imaging system.

### 2.16. *In vivo* pharmacodynamics study

The nude mice bearing PANC-1 cells were divided into four groups of four mice each. Normal saline, free Cur, DHPA-CDB/Cur, and oHA-PBA@DHPA-CDB/Cur (equivalent Cur doses of 20 mg/kg) were administered to the mice every other day (from Day 1 to Day 21), respectively. The body weight and tumor volume of nude mice were measured every other day, and the tumor volume was calculated from the equation: (length × width^2^)/2.

### 2.17. Preliminary histological study

Nude mice were euthanized 3 days after the last treatment administration; subsequently, the tumor tissue was excised, fixed with 4% paraformaldehyde, and embedded in paraffin for sectioning. After H&E staining, the histology of the sections was observed by using a microscope.

## 3. Results and discussion

### 3.1. Characterization of polymers

**Figure 3.**
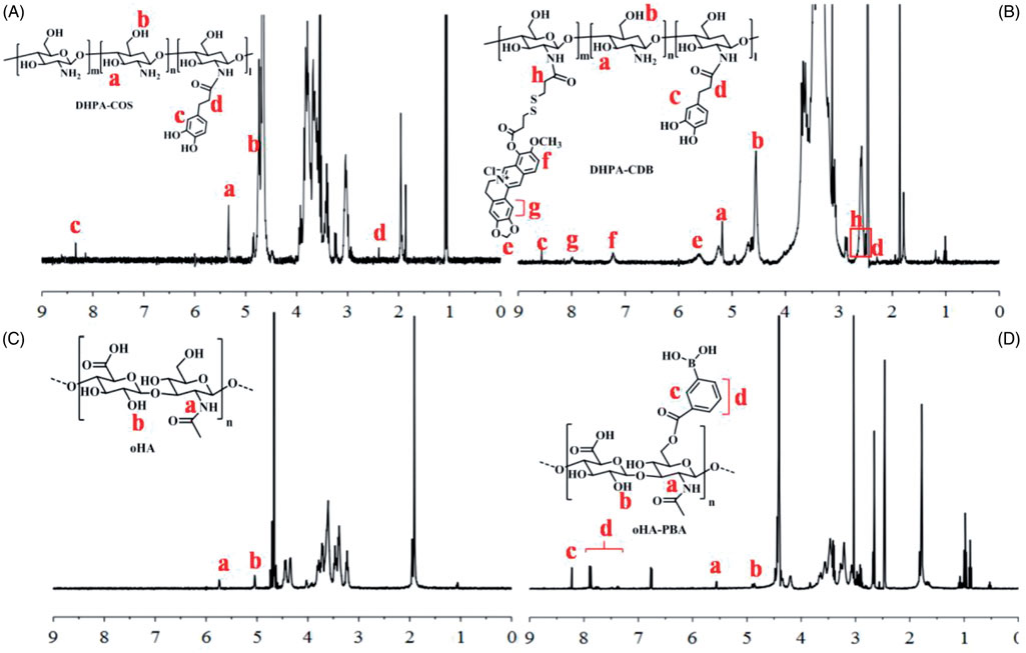
Characterization of polymers by 1 H-NMR: (A) DHPA-COS. (B) DHPA-CDB. (C) oHA. (D) oHA-PBA.

The synthesis of all products was confirmed by ^1 ^H-NMR (Figure 3(A–D)).

#### 3.1.1. Characterization of DHPA-CDB

The ^1 ^H-NMR spectrum of DHPA-COS is shown in Figure 3(A). Two characteristic absorption bands at 4.7 ppm and 5.3 ppm (b, a) were confirmed to result from COS. The signal peaks at 8.3 ppm (c) were due to the benzene ring of DHPA, whereas the signal for –CH_2_– in DHPA was observed at 2.2 ppm (d). It was therefore proved that DHPA was successfully grafted into COS. Therefore, the characteristic peaks of DHPA-CDB were later analyzed. In addition to the characteristic peak of DHPA-COS, new characteristic peaks appeared as a result of the combination of HOOC-SS-Ber (Figure 3(B)). The new peaks at 7.0–8.5 ppm (f, g) corresponded to the rings of Ber. The signal corresponding to –O–CH_2_–O– on Ber was found at 5.6 ppm (e). The signal peaks at 2.5 ppm (h) result from from –CH_2_–, which was located on the side of –S–S–. The above results all proved that the new polymer DHPA-CDB was successfully synthesized.

#### 3.1.2. Characterization of oHA-PBA

The ^1 ^H-NMR spectrum of the oHA raw material is shown in Figure 3(C). In detail, the signals in the range of 1.5–6.0 ppm all belonged to oHA; among them, the peaks at 5.1 ppm (b) and 5.7 ppm were from –OH and –NH–, respectively. In the ^1 ^H-NMR spectrum of oHA-PBA, we successfully identified the characteristic peaks of PBA. As shown in Figure 3(D), the peaks, “c” and “d”, that emerged in the range of 7.3–8.5 ppm were entirely attributable to the benzene ring in PBA.

### 3.2. Characterization of DHPA-CDB/cur and oHA-PBA@DHPA-CDB/cur

**Figure 4.**
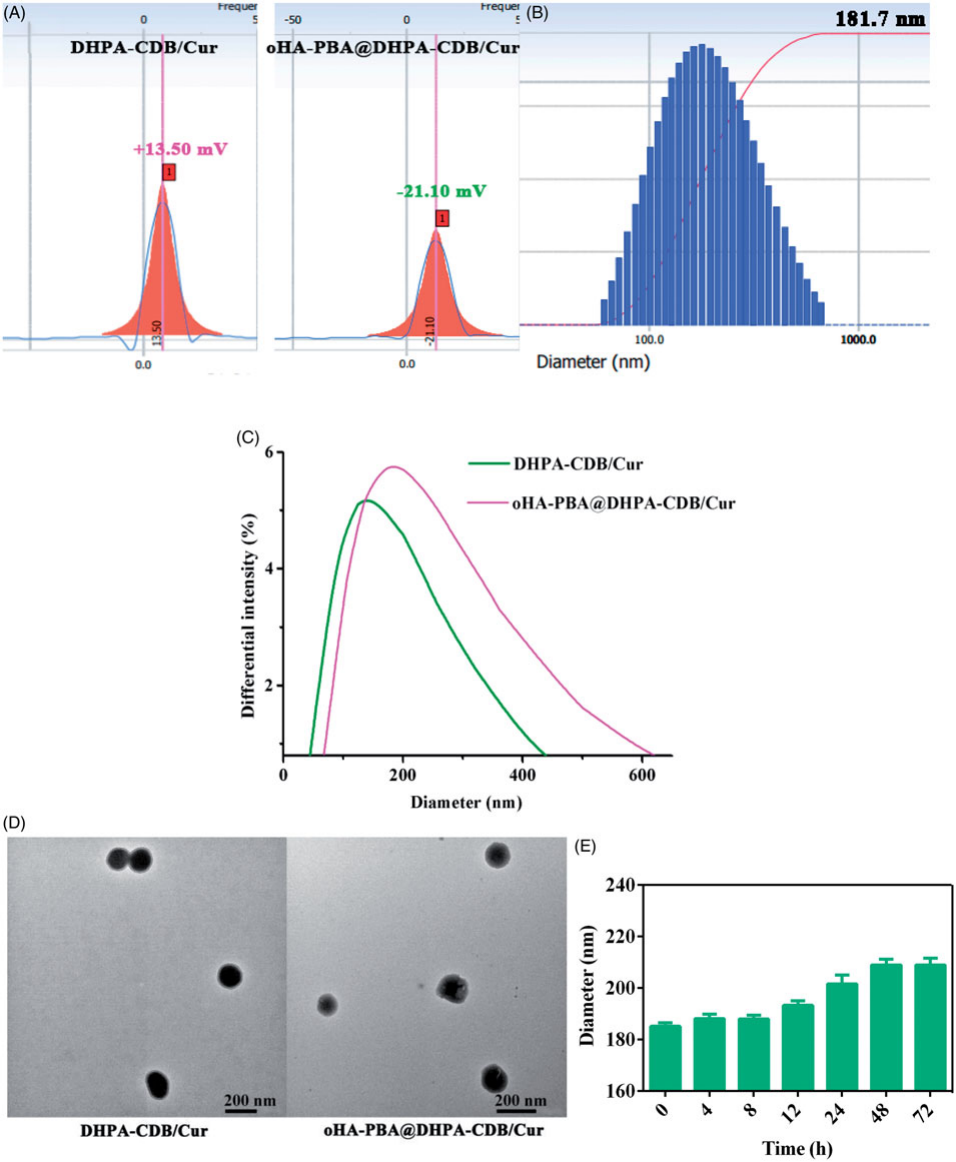
Characterization of DHPA-CDB/Cur and oHA-PBA@DHPA-CDB/Cur: (A) Zeta potential of DHPA-CDB/Cur and oHA-PBA@DHPA-CDB/Cur. (B) Particle size distribution of oHA-PBA@DHPA-CDB/Cur. (C)The change of particle size. (D) TEM image of DHPA-CDB/Cur and oHA-PBA@DHPA-CDB/Cur. (E) The stability of oHA-PBA@DHPA-CDB/Cur in PBS.

DHPA-CDB/Cur was successfully obtained by using the thin film hydration method, and had a zeta potential of +13.5 mV (Figure 4(A)) owing to the existence of COS and Ber. DHPA-CDB/Cur was prepared by coating with negatively charged HA-PBA, and had a zeta potential of −21.10 mV (Figure 4(A)). The average particle size of oHA-PBA@DHPA-CDB/Cur was 181.7 nm; the particle size distribution is shown in Figure 4(B). In addition, as shown in Figure 4(C), the particle size of oHA-PBA@DHPA-CDB/Cur was larger than that of DHPA-CDB/Cur. The changes in the surface charge and particle size indicated that the modification of HA-PBA was successful. The size, PDI, zeta potential, DL, and EE of various Cur preparations are listed in Table 1.

**Table 1.** Characterization of Cur preparations.

Cur preparations	Size (nm)	Zeta (mV)	PDI	DL (%)	EE (%)
DHPA-CDB/Cur	132.4 ± 17.8	+13.5 ± 2.04	0.261 ± 0.09	4.2 ± 0.2	44.1 ± 1.8
oHA-PBA@ DHPA-CDB/Cur	181.7 ± 11.1	−21.1 ± 5.38	0.242 ± 0.14	3.6 ± 0.17	39.8 ± 2.3

The TEM test images of the DHPA-CDB/Cur and oHA-PBA@DHPA-CDB/Cur are shown in Figure 4(D), indicating the spherical structure of the prepared nanoparticles. It was evident from the figure that the size of oHA-PBA@DHPA-CDB/Cur was larger than DHPA-CDB/Cur, which provided further evidence of successful HA-PBA coating.

The electrostatic interactions were unstable under physiological ion conditions; therefore, we investigated the stability of the oHA-PBA@DHPA-CDB/Cur particle size in PBS. As shown in Figure 4(E), the chemical cross-linking of oHA-PBA@DHPA-CDB/Cur was stable and there was no significant change in particle size after 72 h.

### 3.3. Abscission of oHA-PBA

**Figure 5.**
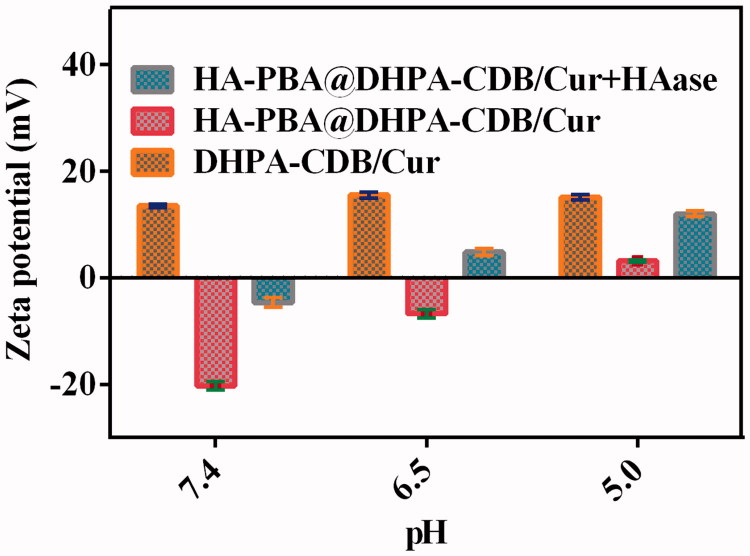
The zeta potential Changes of oHA-PBA@DHPA-CDB/Cur after incubation with HAase at different pH.

As the borate ester bond is highly susceptible to pH changes, we studied the abscission of oHA-PBA at various pH values. The abscission of oHA-PBA from oHA-PBA@DHPA-CDB/Cur was determined from the change in zeta potential after oHA-PBA@DHPA-CDB/Cur incubation at various pH values in the presence or absence of HAase. As shown in Figure 4, the potential of DHPA-CDB/Cur was not significantly changed at different pH conditions. oHA-PBA@DHPA-CDB/Cur transformed to a slightly positive potential as the pH value decreased; this may have been due to the fact that the change in pH led to the abscission of oHA-PBA, but the oHA in the system was not degraded, so the nanoparticles were unstable. In contrast, when the pH value decreased, the zeta potential of oHA-PBA@DHPA-CDB/Cur rapidly changed from negative to positive when exposed to HAase (Figure 5).

### 3.4. Drug release investigation

**Figure 6.**
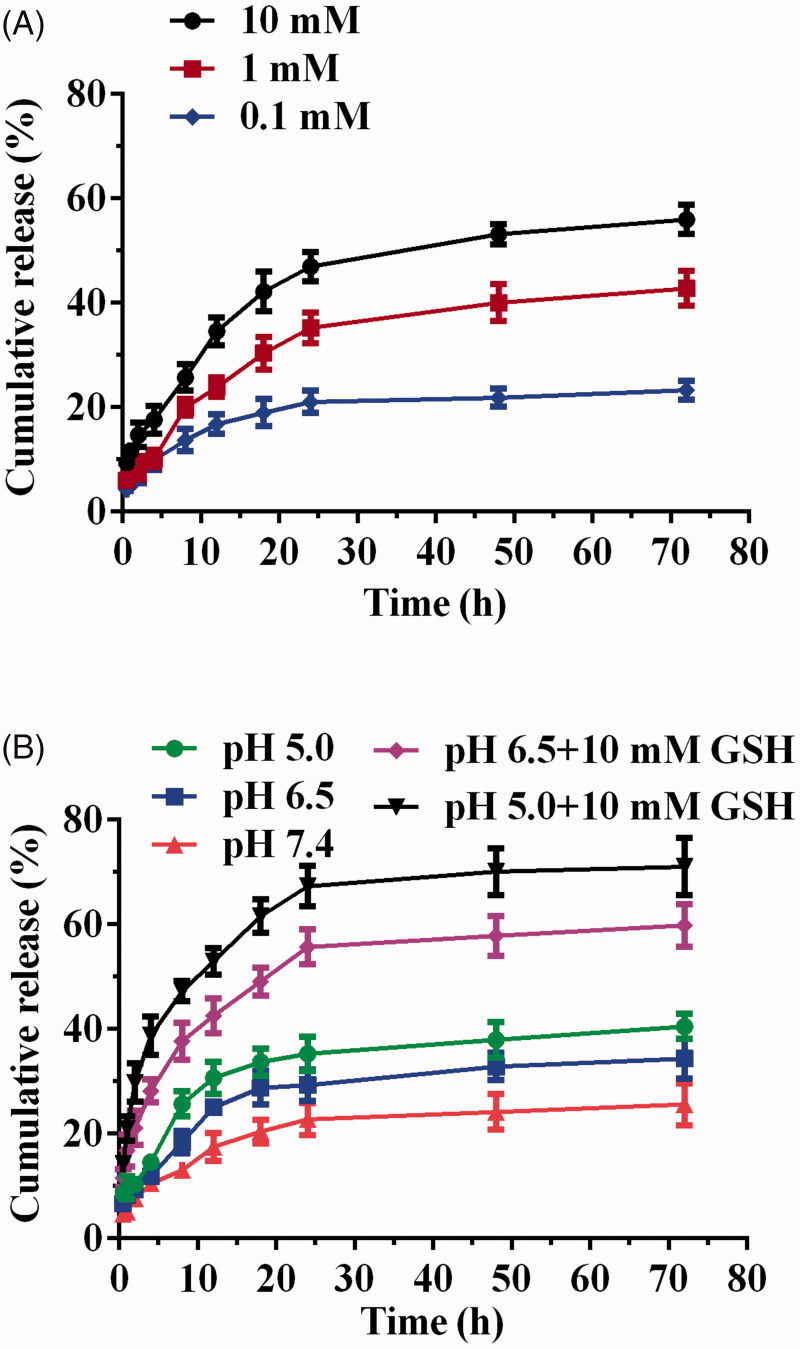
Drug release investigation: (A) GSH-responsive Cur release behavior of DHPA-CDB/Cur. (B) TME-responsive drug release characteristic of oHA-PBA@DHPA-CDB/Cur.

The *in vitro* release of Cur of DHPA-CDB/Cur was examined at different concentrations of GSH (0.1, 1, and 10 mM), and the *in vitro* release curves were plotted (Figure 6(A)). As shown in Figure 6(A), when the concentration of GSH was 0.1 mM, only 23.2% Cur was released, and when the concentration of GSH was increased to 10 mM, 55.9% Cur release was obtained. These results demonstrated that the release of the DHPA-CDB/Cur core has a release profile of the reducing response.

The TME-responsive drug release characteristics were further researched in the release medium (PBS) with different pH values; in addition, PBS containing 10 mM GSH at different pH values was set up to simulate the TME (Figure 6(B)). The results of this experiment showed that Cur was released more rapidly as the pH value was decreased. This may have been due to the cleavage of the pH-responsive boronic ester bond, which led to the loss of the oHA-PBA shell, the destruction of the stable structure of the oHA-PBA@DHPA-CDB/Cur, and enhanced drug release. In the presence of 10 mM GSH, the cumulative release was 59.8% and 71%, respectively. The notable increase in Cur release from oHA-PBA@DHPA-CDB/Cur under acidic conditions with 10 mM GSH suggested that, in the presence of GSH, the disulfide bond of the DHPA-CDB/Cur core was destroyed. The above experiments showed that oHA-PBA@DHPA-CDB/Cur exhibited only minor Cur release in the normal physiological environment (pH 7.4), but that release was increased dramatically in the TME.

### 3.5. Cytotoxicity assay

**Figure 7.**
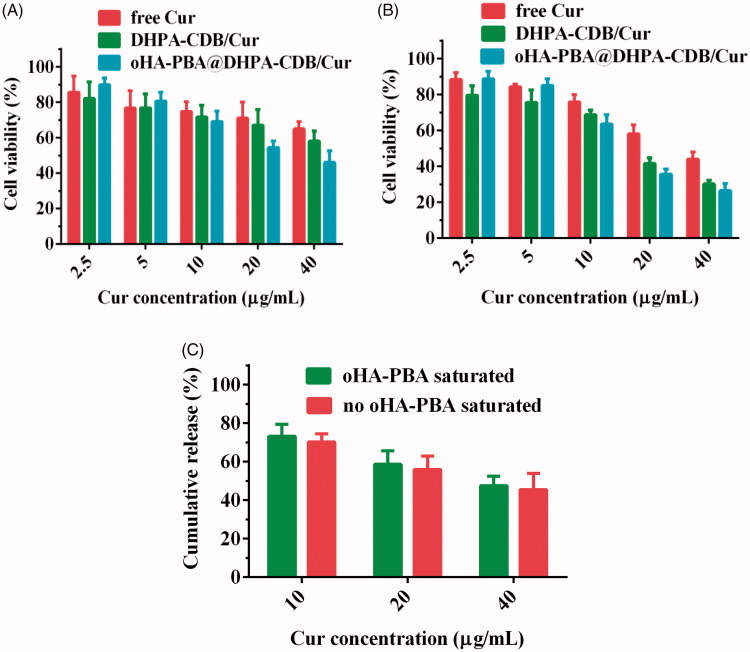
*In vitro* cytotoxicity after 24 h (A) and 48 h (B) with free Cur, DHPA-CDB/Cur, and oHA-PBA@DHPA-CDB/Cur. (C) *In vitro* cytotoxicity of oHA-PBA@DHPA-CDB/Cur in the co-incubation with oHA-PBA.

The cytotoxicity of free Cur and Cur preparation to PANC-1 cells was evaluated by employing the MTT assay (Figure 7(A,B)). As expected, an increase in Cur concentration and the prolongation of Cur action time resulted in a downward trend in the cell survival rate, suggesting that the nanosystems exerted time-dependent and concentration-dependent cytotoxicity. The cytotoxicity of oHA-PBA@DHPA-CDB/Cur was higher than that of free Cur and DHPA-CDB/Cur groups, especially when the concentration was greater than 10 μg/mL; this may have been due to the higher cell internalization and mitochondrial targeting ability of oHA-PBA@DHPA-CDB/Cur, which expedited the intracellular transmission and mitochondrial release of Cur and notably contributed to the enhanced cytotoxicity. Conversely, oHA-PBA@DHPA-CDB/Cur showed the weakest cytotoxicity when the Cur concentration was less than 5 μg/mL; this may have been due to a slight promoting effect of oHA on the growth of PANC-1 cells at low intracellular Cur concentrations.

As expected, after co-incubation with oHA-PBA, oHA-PBA@DHPA-CDB/Cur showed low cytotoxicity compared with untreated cells (Figure 7(C)). This phenomenon indicated that sialic acid epitopes and CD44 receptors on the surface of PANC-1 cells participated in this process and the modification of oHA-PBA could increase the antitumor activity of oHA-PBA@DHPA-CDB/Cur *in vitro*.

### 3.6. *In vitro* cellular uptake and mitochondrial co-localization

**Figure 8.**
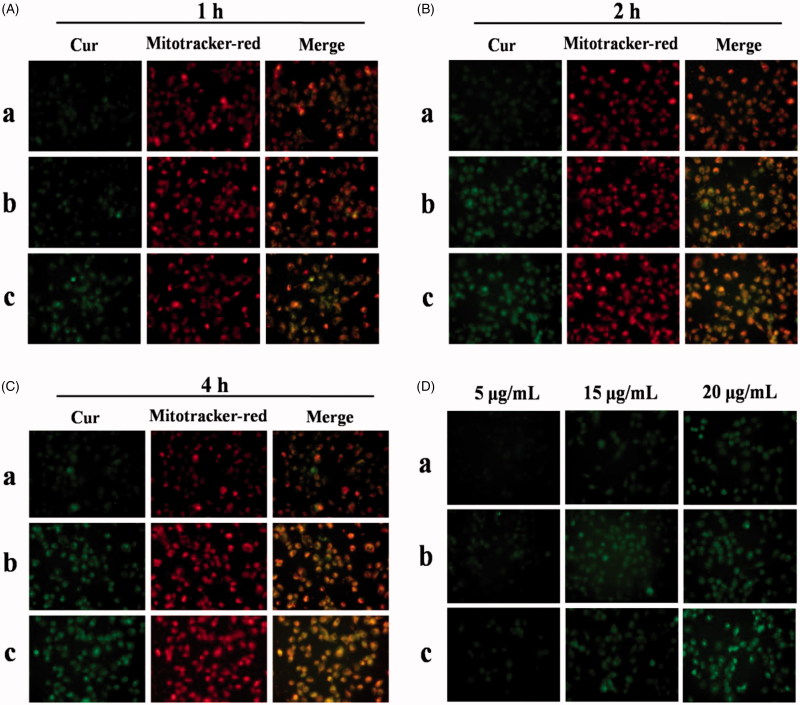
*In vitro* cellular uptake and mitochondrial co-localization of free Cur, DHPA-CDB/Cur, and oHA-PBA@DHPA-CDB/Cur after 1 h (A), 2 h (B), 4 h (C). The mitochondria were stained by Mitotracker-red (red fluorescence). Cur emits green fluorescence itself. Orange fluorescence indicates the overlay between Cur and mitochondria. (D) The concentration-dependent cellular uptake of free Cur, DHPA-CDB/Cur and oHA-PBA@DHPA-CDB/Cur. (a: free Cur, b: DHPA-CDB/Cur, c: oHA-PBA@DHPA-CDB/Cur).

*In vitro* cellular uptake and mitochondrial co-localization of free Cur, DHPA-CDB/Cur, and oHA-PBA@DHPA-CDB/Cur were observed by using an inverted fluorescence microscope. Cur has green fluorescence, and the mitochondria exhibit red fluorescence. As shown in Figure 8(A–C), significant green and orange fluorescence (superposition of green fluorescence and red fluorescence) was observed as the treatment time of PANC-1 cells with DHPA-CDB/Cur and oHA-PBA@DHPA-CDB/Cur was extended, compared with free Cur treatment. oHA-PBA@DHPA-CDB/Cur resulted in high levels of fluorescence intensity in all samples, probably because PBA could act on the sialic acid epitopes on the surface of PANC-1 cells and then entered in the PANC-1 cells through receptor-mediated endocytosis, thereby increasing cellular uptake. The results of the analysis of the above images indicated that oHA-PBA@DHPA-CDB/Cur was clearly taken up into cells and was co-located with the mitochondria. The green fluorescence of Cur was weak in the first hour, but the difference in fluorescence intensity was not significant between 2 h and 4 h, which indicated that the optimum uptake time for the Cur preparations was 2 h–4 h.

PANC-1 cells were separately administered free Cur, DHPA-CDB/Cur, or oHA-PBA@DHPA-CDB/Cur with different equivalent concentrations of Cur. The fluorescence imaging indicated that the uptake of Cur by PANC-1 cells was concentration dependent (Figure 8(D)); in particular, oHA-PBA@DHPA-CDB/Cur showed the maximum cellular uptake. Consequently, we inferred that the optimal uptake concentration of Cur was 20 μg/mL.

### 3.7. Subcellular drug distribution

**Figure 9.**
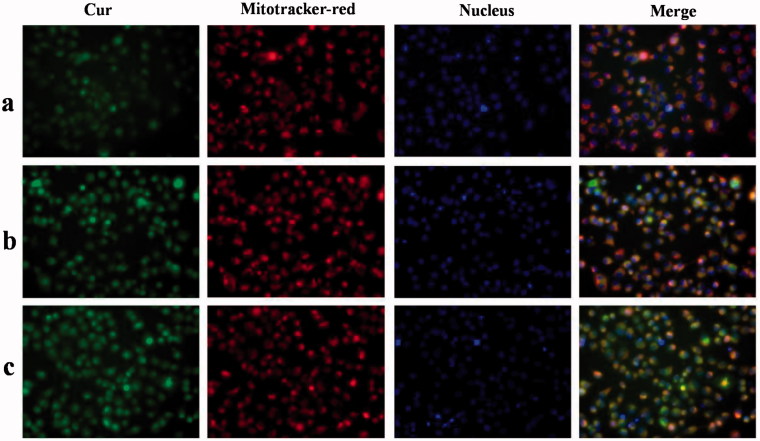
The dynamic distribution of free Cur, DHPA-CDB/Cur, and oHA-PBA@DHPA-CDB/Cur in PANC-1 cells. The mitochondria were stained by Mitotracker-red (red fluorescence). The Nucleus were stained by Hoechst 33342 (blue fluorescence). Cur emits green fluorescence itself. (a: free Cur, b: DHPA-CDB/Cur, c: oHA-PBA@DHPA-CDB/Cur).

In this research, we explored the uptake of Cur preparations and intracellular distribution of Cur from those Cur preparations; the dynamic distribution of Cur in PANC-1 cells in the mitochondria and nucleus was shown in Figure 9. Cur exhibits green fluorescence, the nuclei were stained with blue fluorescence, and the mitochondria were stained with red fluorescence. At 4 h, only a small amount of free Cur was taken up by PANC-1 cells, resulting in weaker green fluorescence. Compared with free Cur, significant green fluorescent was found after the Cur preparation treatmnet. Meanwhile, due to the presence of Ber, Cur preparation groups exhibited significant orange fluorescence, indicating that DHPA-CDB/Cur and oHA-PBA@DHPA-CDB/Cur transported more Cur into PANC-1 cells and preferentially carried Cur to the mitochondria. Further, we observed that most of the green fluorescence appeared outside of the blue fluorescence, suggesting that the Cur preparations entered the cells via caveolar endocytosis.

### 3.8. Mitochondrial membrane depolarization

**Figure 10.**
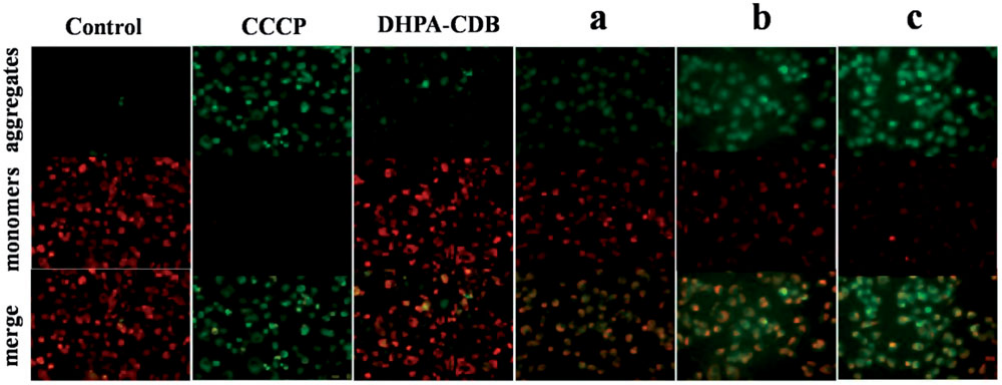
Comparison of Δψm in PANC-1 cells after administered with different groups. (a: free Cur, b: DHPA-CDB/Cur, c: oHA-PBA@DHPA-CDB/Cur).

Apoptosis in the mitochondrial pathway is usually accompanied by a decrease in the Δψm. The change of Δψm was assessed bu using the JC-1 probe, and depolarization of the mitochondrial membrane is represented by a decrease in red fluorescence and an increase in green fluorescence. After CCCP treatment, the Δψm of cells was almost completely lost, which showed strong green fluorescence. In Figure 10, the comparison of Δψm in PANC-1 cells after administration of different treatments in shown. As expected, the positively charged core material (DHPA-CDB) induced a decrease in Δψm, which suggested that DHPA-CDB targeted the mitochondrial membrane potential. In addition, the red fluorescence was more clearly decreased at certain time points in PANC-1 cells treated with oHA-PBA@DHPA-CDB/Cur than those treated with DHPA-CDB/Cur and free Cur. These result suggested that, compared with the DHPA-CDB/Cur and free Cur treatment, oHA-PBA@DHPA-CDB/Cur treatment exerted a stronger effect on Δψm and the initiation of apoptosis in the mitochondrial pathway.

### 3.9. *In vivo* distribution

**Figure 11.**
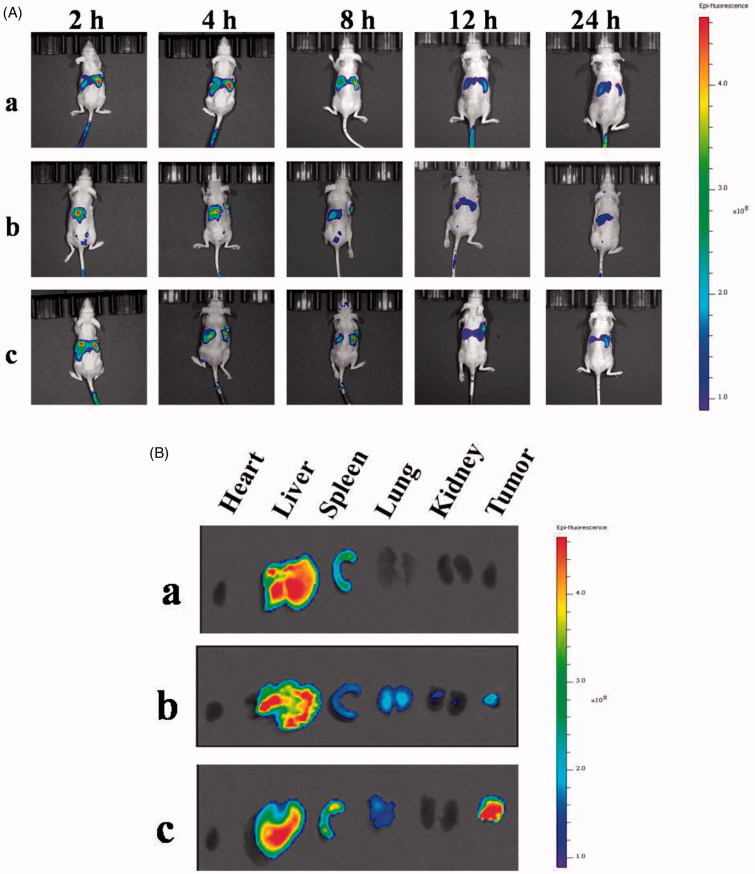
(A) *In vivo* small animal imaging of mice bearing PANC-1 cells with different DiR preparations. (B) The results of the distribution of organs and tumors in mice after 4 h. (a: free DiR, b: DHPA-CDB/DiR, c: oHA-PBA@DHPA-CDB/DiR).

Free DiR, DHPA-CDB/DiR, and oHA-PBA@DHPA-CDB/DiR were injected into nude mice bearing PANC-1 cells via the tail vein. The results of the *in vivo* small animal imaging experiment (Figure 11(A)) demonstrated that oHA-PBA@DHPA-CDB/DiR could effectively accumulate at the tumor site. The CD44 receptor was involved in the formation of tumor blood vessels, so oHA-PBA@DHPA-CDB/DiR had a longer retention time at the tumor site and maintained effective accumulation after 24 h. In contrast, the fluorescence intensity of the untargeted DHPA-CDB/DiR spread to the surrounding tissue and the metabolism was obviously accelerated after 8 h, which may be a result of its instability in the systemic circulation and the ease with which it can be removed by RES. After 4 h, some of the mice were sacrificed and fluorescence imaging of the main organs and tumor was performed. The fluorescence distribution in the organs and tumors of the mice (Figure 11(B)) were consistent with the results of *in vivo* imaging. The fluorescence intensity at the tumor sites, from strong to weak, was in the order oHA-PBA@DHPA-CDB/DiR, DHPA-CDB/DiR, and free DiR.

### 3.10. Pharmacodynamics *in vivo*

**Figure 12.**
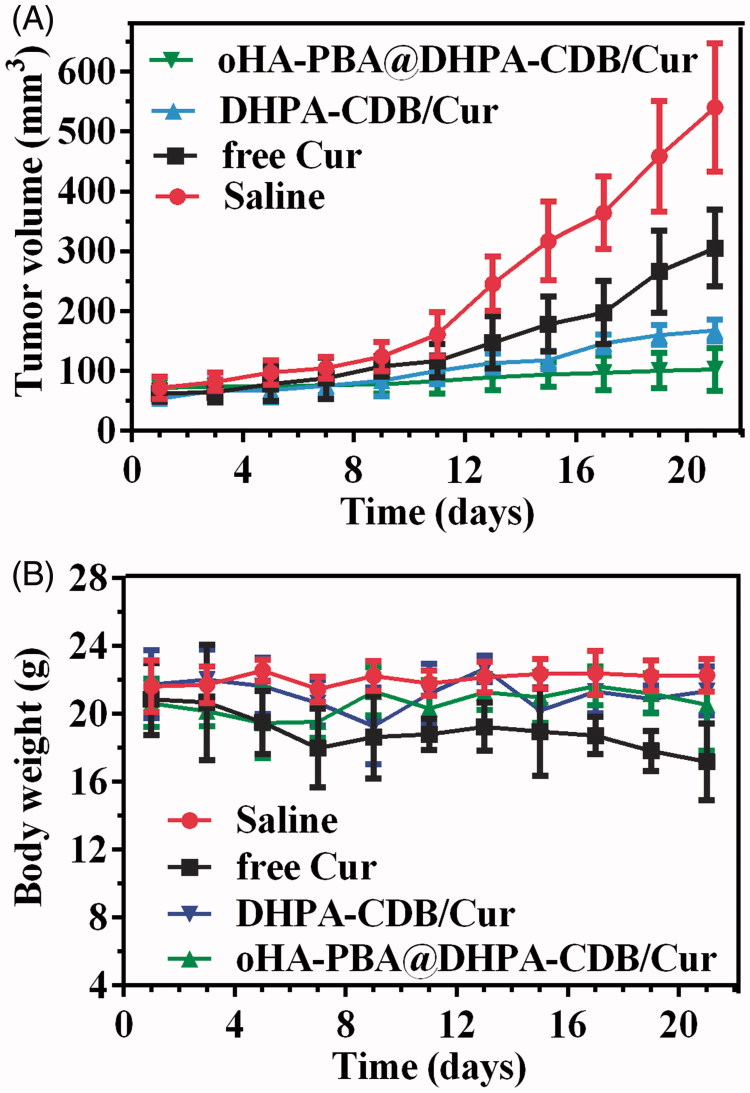
Tumor suppression effect of different Cur preparation *in vivo* based on PANC-1-bearing mice model. Variation of tumor volume (A) and body weight (B) in 21 days.

We established a model of tumor-bearing nude mice to evaluate the antitumor effect of nanoparticles *in vivo*. In Figure 12(A), it is shown that the antitumor effect of the Cur preparation group was significantly higher than that of free Cur and saline. Owing to the targeting and greater physiological stability of oHA-PBA@DHPA-CDB/Cur, the antitumor effect of oHA-PBA@DHPA-CDB/Cur was stronger than that of DHPA-CDB/Cur. In addition, we found that the weight loss of the mice in the free Cur group was the most obvious, and that the mice in the Cur preparation group had smaller changes in body weight (Figure 12(B)). This was attributed to the reduced the systemic toxicity of Cur resulting from its encapsulation in nanoparticles.

### 3.11. Histological analysis

**Figure 13.**
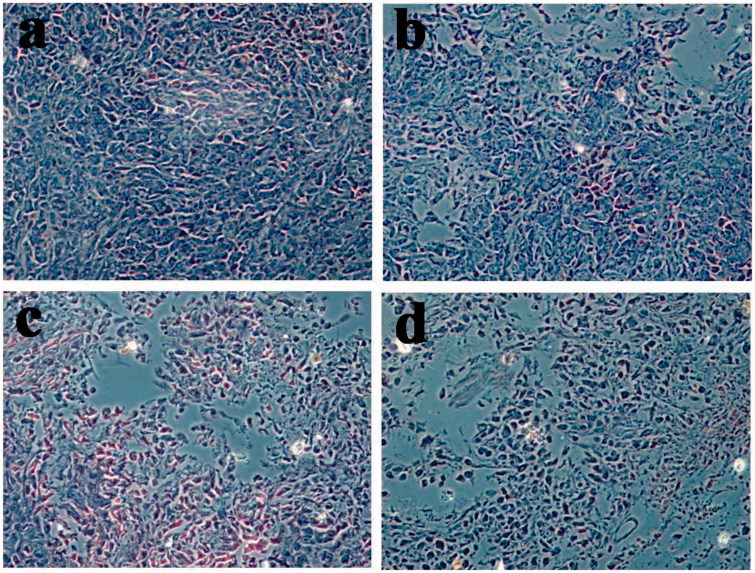
The images of H&E staining. (a: saline b: free Cur, c: DHPA-CDB/Cur, d: oHA-PBA@DHPA-CDB/Cur).

The images of H&E staining are shown in Figure 13. In the corresponding tumor sections, the most obvious tumor pathological changes were observed in oHA-PBA@DHPA-CDB/Cur group compared with the group containing saline, free Cur, and DHPA-CDB/Cur. These experimental results showed the clear advantages of oHA-PBA@DHPA-CDB/Cur, including the most effective induction of tumor apoptosis and cell necrosis. From the *in vivo* antitumor effect experiment, we concluded that oHA-PBA@DHPA-CDB/Cur could improve the therapeutic effect of Cur *in vivo* and prolong blood persistence, thereby reducing the side effects.

## 4. Conclusion

In this study, we prepared polysaccharide nanoparticles for tumor treatment that simultaneously prolonged systemic circulation, mitochondrially targeted drug release, and exhibited TME-responsiveness charge reversal. These nanoparticles achieved the specific targeting and endocytosis of the tumor cells through the specific binding of the sialic acid epitope and the CD44 receptors that are overexpressed on tumor cells. When entering into the TME by receptor-mediated targeting, the boric acid ester bond was broken and oHA was degraded. This nanosystem therefore switched from a positive charge into a negative charge to promote cellular internalization and mitochondrial localization, ultimately achieving GSH-triggered Cur release. Compared with DHPA-CDB/Cur, the prepared oHA-PBA@DHPA-CDB/Cur revealed enhanced cytotoxicity, cellular uptake, and mitochondrial targeting ability in PANC-1 cells by receptor-mediated endocytosis. When oHA-PBA@DHPA-CDB/Cur was effectively localized in the mitochondria, mitochondrial membrane depolarization was also induced. In addition, oHA-PBA@DHPA-CDB/Cur showed the most effective tumor suppression and biosafety, with preferential accumulation by the tumor tissue. Thus, long-circulating oHA-PBA@DHPA-CDB/Cur was successfully prepared with mitochondrial targeting and tumor environment charge reversal capability. This Cur-containing nanosystem, with prolonged blood persistence, effective tumor accumulation, and subsequent subcellular targeting, resulted in significantly improved therapeutic efficacy.

## References

[CIT0001] Babu P, Manu PM, Dhanya TJ, et al. (2017). Bis(3,5-diiodo-2,4,6-trihydroxyphenyl)squaraine photodynamic therapy disrupts redox homeostasis and induce mitochondria-mediated apoptosis in human breast cancer cells. Sci Rep 7:42126. 28169351 10.1038/srep42126 PMC5294812

[CIT0002] Besson E, Gastaldi S, Bloch E, et al. (2019). Embedding cyclic nitrone in mesoporous silica particles for EPR spin trapping of superoxide and other radicals. Analyst 144:4194. 31180410 10.1039/c9an00468h

[CIT0003] Borri C, Centi S, Ratto F, et al. (2018). Polylysine as a functional biopolymer to couple gold nanorods to tumor-tropic cells. J Nanobiotechnol 16:50. 10.1186/s12951-018-0377-7 PMC5984317 29855304

[CIT0004] Chen J, Ding J, Wang Y, et al. (2017). Sequentially responsive shell-stacked nanoparticles for deep penetration into solid tumors. Adv Mater 29: 1701170. 10.1002/adma.201701170 28632302

[CIT0005] Chuang C, Wu PC, Tsai TH, et al. (2017). Development of pH-sensitive cationic PEGylated solid lipid nanoparticles for selective cancer-targeted therapy. J Biomed Nanotechnol 13:192–203. 29377649 10.1166/jbn.2017.2338

[CIT0006] Dai Q, Wilhelm S, Ding D, et al. (2018). Quantifying the ligand-coated nanoparticle delivery to cancer cells in solid tumours. ACS Nano 12:8423. 30016073 10.1021/acsnano.8b03900

[CIT0007] Fan B, Kang L, Chen L, et al. (2017). Systemic siRNA delivery with a dual pH-responsive and tumor-targeted nanovector for inhibiting tumor growth and spontaneous metastasis in orthotopic murine model of breast carcinoma. Theranostics 7:357–76. 28042340 10.7150/thno.16855 PMC5197070

[CIT0008] Gulzar A, Xu J, Wang C, et al. (2019). Tumour microenvironment responsive nanoconstructs for cancer theranostic. Nano Today 26:16–56.

[CIT0009] Hu L, Zhang P, Wang X, et al. (2017). pH-sensitive carboxymethyl chitosan hydrogels via acid-labile ortho ester linkage for potential biomedical applications. Carbohydr Polymers 178:166–79. 10.1016/j.carbpol.2017.09.004 29050582

[CIT0010] Huang Q, Wang L, Yu H, et al. (2019). Advances in phenylboronic acid-based closed-loop smart drug delivery system for diabetic therapy. J Control Release 305:50–64. 31112719 10.1016/j.jconrel.2019.05.029

[CIT0011] Huo C, Xiao K, Zhang S, et al. (2018). H5N1 influenza a virus replicates productively in pancreatic cells and induces apoptosis and pro-inflammatory cytokine response. Front Cell Infect Microbiol 8:386. 30460207 10.3389/fcimb.2018.00386 PMC6232254

[CIT0012] Jeong JY, Hong EH, Lee SY, et al. (2017). Boronic acid-tethered amphiphilic hyaluronic acid derivative-based nanoassemblies for tumor targeting and penetration. Acta Biomater 53:414–26. 28216300 10.1016/j.actbio.2017.02.030

[CIT0013] Jiang D, Mu W, Pang X, et al. (2018). Cascade cytosol delivery of dual-sensitive micelle-tailored vaccine for enhancing cancer immunotherapy. ACS Appl Mater Interfaces 10:37797–811. 30360105 10.1021/acsami.8b09946

[CIT0014] Jing Y, Xiong X, Ming Y, et al. (2018). A multifunctional micellar nanoplatform with pH-triggered cell penetration and nuclear targeting for effective cancer therapy and inhibition to lung metastasis. Adv Healthcare Mater 7:1700974. 10.1002/adhm.201700974 29334189

[CIT0015] Kim J, Lee YM, Kim H, et al. (2016). Phenylboronic acid-sugar grafted polymer architecture as a dual stimuli-responsive gene carrier for targeted anti-angiogenic tumor therapy. Biomaterials 75:102–11. 26491998 10.1016/j.biomaterials.2015.10.022

[CIT0017] Kundu M, Sadhukhan P, Ghosh N, et al. (2019). pH-responsive and targeted delivery of curcumin via phenylboronic acid-functionalized ZnO nanoparticles for breast cancer therapy. J Adv Res 18:161–72. 31032117 10.1016/j.jare.2019.02.036 PMC6479012

[CIT0018] Lang T, Dong X, Zheng Z, et al. (2019). Tumor microenvironment-responsive docetaxel-loaded micelle combats metastatic breast cancer. Sci Bull 64:91–100. 10.1016/j.scib.2018.12.025 36659642

[CIT0019] Ling M, Liu Y, Rao J, et al. (2018). Enhanced tumor retention effect by click chemistry for improved cancer immunochemotherapy. ACS Appl Mater Interfaces 10:17582–17593. 29738228 10.1021/acsami.8b02954

[CIT0020] Lv Q, Yang X, Wang M, et al. (2018). Mitochondria-targeted prostate cancer therapy using a near-infrared fluorescence dye–monoamine oxidase A inhibitor conjugate. J Control Release 279:234. 29679664 10.1016/j.jconrel.2018.04.038

[CIT0021] Ma X, Ren X, Guo X, et al. (2019). Multifunctional iron-based Metal-Organic framework as biodegradable nanozyme for microwave enhancing dynamic therapy. Biomaterials 214:119223. 31174065 10.1016/j.biomaterials.2019.119223

[CIT0022] Pan G, Jia H, Zhu Y, et al. (2018). Cyanine-containing polymeric nanoparticles with imaging/therapy-switchable capability for mitochondria-targeted cancer theranostics. ACS Appl Nano Mater 1:2885.

[CIT0023] Ranalli A, Santi M, Capriotti L, et al. (2017). Peptide-based stealth nanoparticles for targeted and pH-triggered delivery. Bioconjugate Chem 28:627. 10.1021/acs.bioconjchem.6b00701 28107619

[CIT0024] Roma-Rodrigues C, Pombo I, Raposo L, et al. (2019). Nanotheranostics targeting the tumor microenvironment. Front Bioeng Biotechnol 7:197. 31475143 10.3389/fbioe.2019.00197 PMC6703081

[CIT0025] Seidi K, Neubauer HA, Moriggl R, et al. (2018). Tumor target amplification: implications for nano drug delivery systems. J Control Release 275:142–61. 29454742 10.1016/j.jconrel.2018.02.020

[CIT0026] Song H, Wang C, Zhang H, et al. (2019). A high-loading drug delivery system based on magnetic nanomaterials modified by hyperbranched phenylboronic acid for tumor-targeting treatment with pH response. Colloids Surf B Biointerfaces 182:110375. 31351268 10.1016/j.colsurfb.2019.110375

[CIT0027] Song J, Lin C, Yang X, et al. (2019). Mitochondrial targeting nanodrugs self-assembled from 9-O-octadecyl substituted berberine derivative for cancer treatment by inducing mitochondrial apoptosis pathways. J Control Release 294:27–42. 30445003 10.1016/j.jconrel.2018.11.014

[CIT0028] Sun C, Cao Y, Zhu P, et al. (2017). A mitochondria-targeting artemisinin derivative with sharply increased antitumor but depressed anti-yeast and anti-malaria activities. Sci Rep 7:45665. 28368011 10.1038/srep45665 PMC5377301

[CIT0029] Taleb M, Ding Y, Wang B, et al. (2019). Dopamine delivery via pH-sensitive nanoparticles for tumor blood vessel normalization and an improved effect of cancer chemotherapeutic drugs. Adv Healthcare Mater 8:1900283. 10.1002/adhm.201900283 31379139

[CIT0030] Tan Y, Zhu Y, Zhao Y, et al. (2018). Mitochondrial alkaline pH-responsive drug release mediated by celastrol loaded glycolipid-like micelles for cancer therapy. Biomaterials 154:169–81. 29128845 10.1016/j.biomaterials.2017.07.036

[CIT0031] Tuo J, Xie Y, Song J, et al. (2016). Development of a novel berberine-mediated mitochondria-targeting nano-platform for drug-resistant cancer therapy. J Mater Chem B 4:6856–64. 10.1039/c6tb01730d 32263579

[CIT0032] Wang S, Zhang J, Wang Y, et al. (2016). Hyaluronic acid-coated PEI-PLGA nanoparticles mediated co-delivery of doxorubicin and miR-542-3p for triple negative breast cancer therapy. Nanomedicine 12:411–20. 26711968 10.1016/j.nano.2015.09.014

[CIT0033] Wang Y, Xie Y, Li J, et al. (2017). Tumor-penetrating nanoparticles for enhanced anticancer activity of combined photodynamic and hypoxia-activated therapy. ACS Nano 2:2227–38. 10.1021/acsnano.6b08731 PMC5332348 28165223

[CIT0034] Wang Y, Yang M, Qian J, et al. (2019a). Sequentially self-assembled polysaccharide-based nanocomplexes for combined chemotherapy and photodynamic therapy of breast cancer. Carbohydr Polym 203:203–13. 30318205 10.1016/j.carbpol.2018.09.035

[CIT0035] Wang Y, Zhang T, Hou C, et al. (2019b). Mitochondria-specific anticancer drug delivery based on reduction-activated polyprodrug for enhancing the therapeutic effect of breast cancer chemotherapy. ACS Appl Mater Interfaces 11:29330–40. 31329411 10.1021/acsami.9b10211

[CIT0036] Xu X, Saw PE, Tao W, et al. (2017). Tumor microenvironment-responsive multistaged nanoplatform for systemic RNAi and cancer therapy. Nano Lett 17:4427–35. 28636389 10.1021/acs.nanolett.7b01571 PMC5615408

[CIT0037] Xue Y, Tian J, Xu L, et al. (2019). Ultrasensitive redox-responsive porphyrin-based polymeric nanoparticles for enhanced photodynamic therapy. Eur Polym J 110:344–54.

[CIT0038] Yang G, Xu L, Xu J, et al. (2018). Smart nanoreactors for pH-responsive tumor homing, mitochondria-targeting, and enhanced photodynamic-immunotherapy of cancer. Nano Lett 18:2475. acs.nanolett.8b00040. 29565139 10.1021/acs.nanolett.8b00040

[CIT0039] Zhang Y, Cai K, Li C, et al. (2018). Macrophage-membrane-coated nanoparticles for tumor-targeted chemotherapy. Nano Lett 18:1908–15. 29473753 10.1021/acs.nanolett.7b05263 PMC7470025

[CIT0040] Zhang Z, Huang M, Xu W, et al. (2019). Stimulus-responsive nanoscale delivery systems triggered by the enzymes in the tumor microenvironment. Eur J Pharm Biopharm 137:122–30. 30776412 10.1016/j.ejpb.2019.02.009

[CIT0041] Zheng Y, Lu H, Zhu J, et al. (2017). A low-power white light triggered AIE polymer nanoparticles with high ROS quantum yield for mitochondria-targeted and image-guided photodynamic therapy. J Mater Chem B 5:6277. 10.1039/c7tb01443k 32264443

[CIT0042] Zhou H, Fu C, Chen X, et al. (2018). Mitochondria-targeted zirconium metal-organic frameworks for enhancing the efficacy of microwave thermal therapy against tumors. Biomater Sci 6:1535. 29670952 10.1039/c8bm00142a

[CIT0043] Zhou Q, Hou Y, Li Z, et al. (2017). Dual-pH sensitive charge-reversal nanocomplex for tumor-targeted drug delivery with enhanced anticancer activity. Theranostics 7:1806–19. 28638469 10.7150/thno.18607 PMC5479270

[CIT0044] Zhou Z, Zhang M, Liu Y, et al. (2018). Reversible covalent cross-linked polycations with enhanced stability and ATP-responsive behavior for improved siRNA delivery. Biomacromolecules 19:3776–87. 30081638 10.1021/acs.biomac.8b00922

